# Supplementary material for: Changes in leisure-time physical activity during the adult life span and relations to cardiovascular risk factors—Results from multiple Swedish studies
Source: PLoS One. 2021 Aug 19;16(8):e0256476. doi: 10.1371/journal.pone.0256476 (PMC8375969; doi:10.1371/journal.pone.0256476)
Supplement: S1 Table — (DOCX) [file pone.0256476.s001.docx]

| Study | N of PA categories | Definition |
| --- | --- | --- |
| ULSAM | 4 | 1. Mainly sedentary behavior. 2. Walking or cycling for pleasure. 3. Recreational sports or heavy gardening at least 3 hours every week. 4. Regularly engage in hard physical training |
| PIVUS | 4 | Two questions were used: How many times a week do you engage in light activity for 30 min? How many times a week do you engage in hard exercise for 30 min? From these two questions four groups were defined: 1. LIght activity only, < 2 times a week. 2. LIght activity only, > 1 times a week. 3. Hard exercise 1 or 2 times a week. 4. Hard exercise > 2 times a week. |
| EpiHealth | 5 | A visual analogue scale ranging from 1-5, where 1 was defined as “Mainly sitting”, 3 was defined as “Walking 30 min a day” and 5 was defined as “Hard exercise 30 min a day” |
| SHE | 4 | 1. Mainly sedentary behavior. 2. Light activity >3h a week. 3. Medium exercise >2h a week. 4. Hard exercise several times a week. |
| SHM | 4 | Same as in SHE |
