# Supplementary material for: Changes in leisure-time physical activity during the adult life span and relations to cardiovascular risk factors—Results from multiple Swedish studies
Source: PLoS One. 2021 Aug 19;16(8):e0256476. doi: 10.1371/journal.pone.0256476 (PMC8375969; doi:10.1371/journal.pone.0256476)
Supplement: S2 Table — The regression coefficient (Beta) vs PA is given. (DOCX) [file pone.0256476.s002.docx]

| **EpiHealth** | | | | |
| --- | --- | --- | --- | --- |
| Variable | Age-group | Beta | SE | p-value |
| TG | 45 | -.171 | .013 | 1.310e-41 |
| TG | 55 | -.165 | .012 | 7.359e-40 |
| TG | 65 | -.137 | .012 | 5.017e-31 |
| Glucose | 45 | -.047 | .013 | 1.563e-06 |
| Glucose | 55 | -.066 | .011 | 4.286e-09 |
| Glucose | 65 | -.054 | .012 | 4.315e-06 |
| SBP | 45 | -.032 | .011 | .00417 |
| SBP | 55 | -.024 | .012 | .045 |
| SBP | 65 | -.008 | .013 | .52 |
| BMI | 45 | -.195 | .013 | 2.067e-53 |
| BMI | 55 | -.201 | .012 | 1.844e-59 |
| BMI | 65 | -.183 | .012 | 3.135e-52 |
| LDL | 45 | -.068 | .012 | 9.288e-09 |
| LDL | 55 | -.039 | .012 | .00094 |
| LDL | 65 | .002 | .012 | .88 |
| HDL | 45 | .169 | .011 | 4.424e-52 |
| HDL | 55 | .142 | .012 | 9.577e-34 |
| HDL | 65 | .138 | .011 | 8.816e-33 |
|  | | | | |
| **ULSAM** | | | | |
| Variable | Time | Beta | SE | p-value |
| TG | 50 | -.123 | .026 | 3.096e-06 |
| TG | 60 | -.075 | .026 | .0032 |
| TG | 70 | -.121 | .048 | .011 |
| TG | 77 | -.054 | .052 | .298 |
| TG | 82 | -.132 | .057 | .021 |
| Glucose | 50 | -.023 | .022 | .29 |
| Glucose | 60 | -.052 | .029 | .082 |
| Glucose | 70 | -.091 | .041 | .025 |
| Glucose | 77 | -.028 | .046 | .54 |
| Glucose | 82 | -.063 | .056 | .26 |
| SBP | 50 | -.018 | .023 | .43 |
| SBP | 60 | -.047 | .033 | .15 |
| SBP | 70 | .013 | .041 | .75 |
| SBP | 77 | .039 | .052 | .45 |
| SBP | 82 | .099 | .052 | .056 |
| BMI | 50 | -.048 | .026 | .062 |
| BMI | 60 | -.101 | .033 | .0021 |
| BMI | 70 | -.055 | .045 | .21 |
| BMI | 77 | .009 | .051 | .85 |
| BMI | 82 | -.056 | .06 | .35 |
| LDL | 50 | -.053 | .029 | .062 |
| LDL | 60 | .031 | .022 | .11 |
| LDL | 70 | .072 | .035 | .037 |
| LDL | 77 | .034 | .037 | .36 |
| LDL | 82 | .008 | .042 | .83 |
| HDL | 50 | .061 | .033 | .060 |
| HDL | 60 | .091 | .026 | .00058 |
| HDL | 70 | .137 | .048 | .0039 |
| HDL | 77 | .135 | .051 | .0071 |
| HDL | 82 | -.005 | .052 | .92 |
|  | | | | |
| **PIVUS** | | | | |
| Variable | Time | Beta | SE | p-value |
| TG | 70 | -.107 | .043 | .012 |
| TG | 75 | -.118 | .049 | .016 |
| TG | 80 | -.075 | .061 | .21 |
| Glucose | 70 | -.013 | .034 | .69 |
| Glucose | 75 | -.099 | .044 | .013 |
| Glucose | 80 | -.052 | .047 | .27 |
| SBP | 70 | -.064 | .044 | .15 |
| SBP | 75 | .031 | .045 | .50 |
| SBP | 80 | .033 | .057 | .56 |
| BMI | 70 | -.093 | .041 | .025 |
| BMI | 75 | -.069 | .049 | .155 |
| BMI | 80 | -.184 | .063 | .0037 |
| LDL | 70 | .057 | .039 | .14 |
| LDL | 75 | .017 | .046 | .71 |
| LDL | 80 | .128 | .053 | .015 |
| HDL | 70 | .121 | .042 | .00225506 |
| HDL | 75 | .089 | .048 | .06252566 |
| HDL | 80 | -.001 | .052 | .98104154 |
