# Supplementary material for: Changes in leisure-time physical activity during the adult life span and relations to cardiovascular risk factors—Results from multiple Swedish studies
Source: PLoS One. 2021 Aug 19;16(8):e0256476. doi: 10.1371/journal.pone.0256476 (PMC8375969; doi:10.1371/journal.pone.0256476)
Supplement: S3 Table — The regression coefficient (Beta, log odds) vs PA is given. (DOCX) [file pone.0256476.s003.docx]

| **EpiHealth** |  |  |  |  |  |
| --- | --- | --- | --- | --- | --- |
| Variable | | Age | Beta | SE | p-value |
| MetS | | 45 | -.417 | .037 | 3.071e-29 |
| MetS | | 55 | -.362 | .032 | 2.250e-29 |
| MetS | | 65 | -.379 | .03 | 1.521e-36 |
| MetScomponents | | 45 | -.354 | .024 | 1.050e-48 |
| MetScomponents | | 55 | -.33 | .024 | 6.880e-43 |
| MetScomponents | | 65 | -.359 | .024 | 6.559e-52 |
|  |  |  |  |  |  |
| **ULSAM** |  |  |  |  |  |
| Variable | | Age | Beta | SE | p-value |
| MetS | | 50 | -.186 | .087 | .03230367 |
| MetS | | 60 | -.261 | .112 | .01975011 |
| MetS | | 70 | -.364 | .126 | .00381275 |
| MetS | | 77 | -.27 | .141 | .05596719 |
| MetS | | 82 | -.216 | .146 | .13915574 |
| MetScomponents | | 50 | -.102 | .052 | .04935329 |
| MetScomponents | | 60 | -.259 | .066 | .0000902 |
| MetScomponents | | 70 | -.387 | .085 | 5.720e-06 |
| MetScomponents | | 77 | -.212 | .097 | .02812009 |
| MetScomponents | | 82 | -.216 | .11 | .04960597 |
|  |  |  |  |  |  |
| **PIVUS** |  |  |  |  |  |
| Variable | | Age | Beta | SE | p-value |
| MetS | | 70 | -.502 | .121 | .00003431 |
| MetS | | 75 | -.347 | .118 | .00319633 |
| MetS | | 80 | -.168 | .148 | .2571646 |
| MetScomponents | | 70 | -.477 | .083 | 1.103e-08 |
| MetScomponents | | 75 | -.335 | .094 | .00037453 |
| MetScomponents | | 80 | -.281 | .12 | .01879243 |
