# Supplementary material for: Changes in leisure-time physical activity during the adult life span and relations to cardiovascular risk factors—Results from multiple Swedish studies
Source: PLoS One. 2021 Aug 19;16(8):e0256476. doi: 10.1371/journal.pone.0256476 (PMC8375969; doi:10.1371/journal.pone.0256476)
Supplement: Attachment — Submitted filename: Plos One Review-May 21 (2021).docx [file pone.0256476.s004.docx]

**PLOS ONE:** Changes in leisure-time physical activity during the adult life span and relations to cardiovascular risk factors - results from multiple Swedish studies

**Manuscript number:** PONE-D-21-14877

The present study analyzes trends in leisure time physical activity (LTPA) and cardiovascular disease risk factors from multiple studies in Sweden. The authors identify significant increases in LTPA across the lifespan and demonstrate the LTPA was associated with an improved cardiovascular risk profile.

**Minor Comments**

Line 28: The authors mention the debate about the optimal diet and alcohol intake for CVD yet this isn’t evaluated in the paper.

Line 32: Since the authors mention genetic contributors to PA and fitness, the paper could be strengthened by providing heritability estimates for physical activity, fitness and CVD, as well as mentioning any shared heritability.

Line 41: The authors state that the links between PA and CVD in different age groups is “poorly studied” yet there is a plethora of literature on this topic. If the authors have reason to think that there are age-dependent effects of PA on CVD risk factors, more evidence should be provided to justify this research question.

Line 51: Including the sample sizes of each study in the methods would be informative for the reader.

Line 93: Are there issues with including overnight fasts with a 6 hour fast? If so, a sensitivity analysis should be considered.

**Major Comments**

Overall the introduction needs to be significantly revised to outline the current evidence on the topic and specify the research gap that this study is addressing.

The authors outline several statistical tests in each study that adjust for a variety of confounders and use different statistical methods. This makes it unclear to the reader what the primary objectives of the study are and how the results can be compared across studies. Without providing a more succinct analysis, such as performing a pooled analysis, the findings do not sufficiently support the conclusions of the study. In addition, many statistical tests are outlined in the methods without any adjustment for multiple testing.

Issues with data harmonization should be discussed. The anchors of each scale are quite different for some measures (e.g., 5= hard exercise > 2 days per week in PIVUS and 5= hard exercise 30 minutes per day in EpiHealth). The implications of this harmonization and potential measurement error should be discussed.
